# Outcomes of a Caregiver-Focused Short Message Service (SMS) Intervention to Reduce Intake of Sugar-Sweetened Beverages in Rural Caregivers and Adolescents

**DOI:** 10.3390/nu15081957

**Published:** 2023-04-19

**Authors:** Donna-Jean P. Brock, Maryam Yuhas, Kathleen J. Porter, Philip I. Chow, Lee M. Ritterband, Deborah F. Tate, Jamie M. Zoellner

**Affiliations:** 1 Department of Public Health Sciences, Community-Based Health Equity Research Program, University of Virginia, Christiansburg, VA 24073, USA; dpb2n@virginia.edu (D.-J.P.B.);; 2 Department of Nutrition and Food Studies, Falk College, Syracuse University, Syracuse, NY 13244, USA; myuhas@syr.edu; 3 Center for Behavioral Health and Technology, Department of Psychiatry and Neurobehavioral Sciences, University of Virginia, Charlottesville, VA 22908, USA; pic2u@virginia.edu (P.I.C.);; 4 Department of Nutrition, Communication for Health Applications and Interventions Core, University of North Carolina, Chapel Hill, NC 27599, USA; dtate@unc.edu

**Keywords:** short message service, school-based caregiver intervention, sugar-sweetened beverages, rural population

## Abstract

This study examined enrollment, retention, engagement, and behavior changes from a caregiver short message service (SMS) component of a larger school-based sugar-sweetened beverage (SSB) reduction intervention. Over 22 weeks, caregivers of seventh graders in 10 Appalachian middle schools received a two-way SMS Baseline Assessment and four monthly follow-up assessments to report their and their child’s SSB intake and select a personalized strategy topic. Between assessments, caregivers received two weekly one-way messages: one information or infographic message and one strategy message. Of 1873 caregivers, 542 (29%) enrolled by completing the SMS Baseline Assessment. Three-quarters completed Assessments 2–5, with 84% retained at Assessment 5. Reminders, used to encourage adherence, improved completion by 19–40%, with 18–33% completing after the first two reminders. Most caregivers (72–93%) selected a personalized strategy and an average of 28% viewed infographic messages. Between Baseline and Assessment 5, daily SSB intake frequency significantly (*p* < 0.01) declined for caregivers (−0.32 (0.03), effect size (ES) = 0.51) and children (−0.26 (0.01), ES = 0.53). Effect sizes increased when limited to participants who consumed SSB twice or more per week (caregivers ES = 0.65, children ES = 0.67). Findings indicate that an SMS-delivered intervention is promising for engaging rural caregivers of middle school students and improving SSB behaviors.

## 1. Introduction

Across age cohorts, adolescents are among the highest sugar-sweetened beverage (SSB) consumers in the United States, with an average daily intake of around 200 kilocalories (kcals) [1]. In the health disparate and largely rural Southwestern Virginia region of Appalachia, adolescent SSB intake is around 470 kcals per day, or twice the national level [2]. Likewise, data have shown that adults in the region consume about 475 kcals of SSB per day [3]. High SSB consumption has been consistently linked to adverse health outcomes, such as obesity, type 2 diabetes, cardiovascular disease, and dental carries, making preventing or reducing adolescent intake an important public health priority, especially in Appalachia [4,5,6,7]. Because high consuming adults can influence adolescent SSB behaviors through role modeling and control of the home environment, there is a need for multi-level interventions that include caregivers [8,9,10]. However, engaging caregivers and adolescents in rural areas is challenging given the many barriers to accessing in-person programing, including lack of transportation and natural geographical dispersion [11]. While school-based programming provides an opportunity to reach a large and representative sample of adolescents in rural regions, the difficulty in engaging their caregivers remains. Interventions that use technology, such as messages delivered by short message service (SMS), to engage caregivers may help decrease health inequities by providing a solution to accessibility issues in rural communities [12,13,14].

In general, interventions using SMS for caregivers of children and adolescents have been found to be acceptable, engaging, and effective at improving a variety of childhood health behaviors [15,16,17,18]. However, SMS health interventions in rural areas of the United States are limited to small-scale pilot and feasibility studies [19,20]. While historically rural regions have experienced a “digital divide” that has hindered implementing mobile health interventions, technological advances have shrunk this divide, especially related to mobile phone use [21,22]. In fact, many schools across the United States have adopted SMS-based communication systems for parents [23]. This opens an opportunity for greater study of school-based SMS interventions that target caregivers of adolescents in rural communities.

Studies on effective practices for SMS health interventions are often limited in their generalizability due to the specificity of the targeted behavior. Regardless, there is some consensus on the importance of message tailoring and personalization, provision of feedback on behavioral outcomes via two-way assessments, and use of reminders to engage with assessments [12,24,25]. Furthermore, a meta-analysis of mobile health interventions targeting children and adolescents indicated that effects are strengthened when caregivers are included [26]. Specific to SSB reduction interventions, a pilot study by Zahid and Riecks provides evidence that an SMS program targeting parents of children aged six to twelve years could improve home availability and parent and child consumption of SSBs [27]. Systematic reviews reveal a lack of school-based trials that used technology to engage caregivers in SSB reduction, resulting in a gap in knowledge for which engagement practices might be most effective [28,29].

The caregiver SMS intervention component of the larger school-based Kids SIPsmartER study aims to improve SSB behaviors in rural Appalachian middle schoolers and their caregivers [30]. The intervention uses evidence-based practices to engage caregivers, such as the use of two-way messaging, assessment with feedback messages, personalization of strategies, and educational infographics. This offers the opportunity to address the gap in the literature by analyzing a text message SSB reduction intervention for caregivers of adolescents who live in rural communities. The objectives of this study are to explore caregiver process and outcome data for enrollment and retention, engagement with SMS, and behavioral changes in SSB consumption for caregivers and their children.

## 2. Materials and Methods

### 2.1. Study Description

This study is focused on the caregiver component of the larger Kids SIPsmartER study. The study protocol is registered with clinicaltrials.gov (NCT03740113) and details of Kids SIPsmartER trial methodology have been published [30]. In brief, the intervention targeted seventh grade students and their caregivers. Kids SIPsmartER is grounded in the theory of planned behavior and incorporates health, media, numeracy, and public health literacy concepts. The study was a five-year cluster randomized controlled trial of 12 schools in which four schools were recruited each year and randomized into intervention or control. In the first year that a school participated in the intervention, research coordinators led implementation of the 12-lesson classroom curriculum for students. Schools could continue to participate in the study for a second and third year in which the teachers transitioned into leading the lesson implementation and research coordinators provided technical assistance and support. In conjunction with the classroom lessons, caregivers with seventh grade children in any of these three years of school participation had the option to join a concurrent SMS component of the study. One caregiver per seventh grade student was eligible to participate, regardless of SSB behaviors. The caregiver SMS component was consistently managed by the research team throughout all years of a school’s participation.

This study includes the first four years of data from the Kids SIPsmartER caregiver SMS intervention. In total, caregiver data was collected across 17 cohorts in 10 Appalachian middle schools. Of these cohorts, 10 were during a school’s first year of receiving the intervention and seven in the second year. Eight of the schools were in Southwest Virginia and two in Southern West Virginia.

This study was approved by the University of Virginia Institutional Review Board and caregivers provided informed consent. Only consented caregivers during years in which schools participated in the SMS intervention were included in the analysis. Schools in the third year of implementation are not included due to COVID-19 interruptions and substantially different caregiver enrollment procedures that are beyond the scope of this study to explore.

### 2.2. Recruitment and Enrollment Procedures

**Figure 1:**
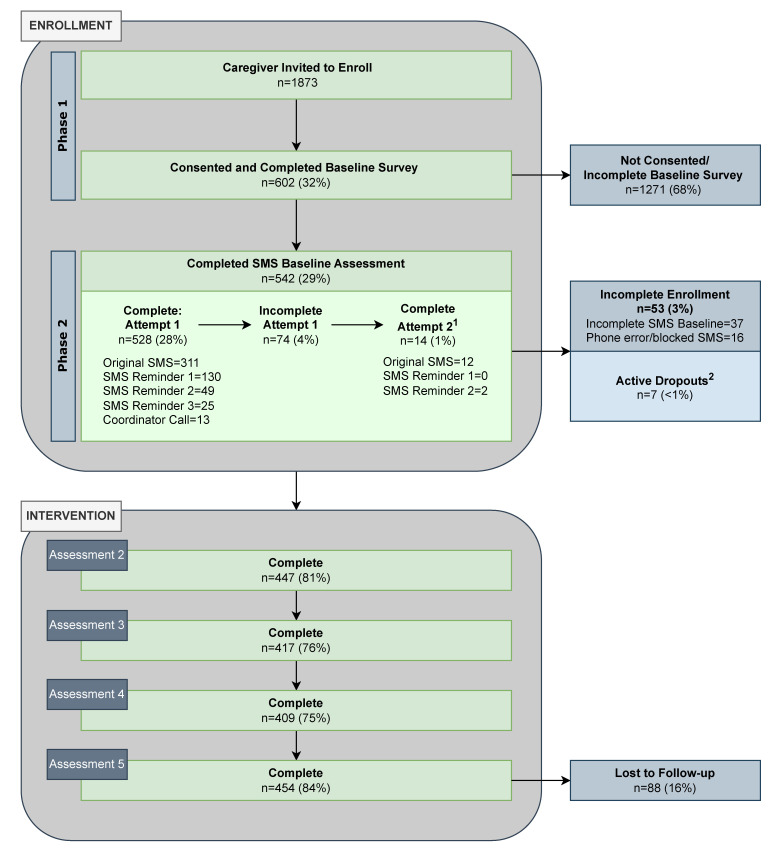
Kids SIPsmartER caregiver SMS consort diagram. ^1^ Non-responders at SMS Baseline Assessment, Attempt 1 (*n* = 74) are not considered fully enrolled. They receive a second attempt to complete this assessment and enroll in the intervention at the same time enrolled caregivers receive Assessment 2. For the 14 caregivers that responded at Attempt 2, SSB intake data was carried forward for Assessment 2. ^2^ Active dropout reflects a caregiver request to stop SMS messages (although caregivers could request to stop receiving SMS at any time during the intervention, none did so past Cycle 2, active dropouts were removed from analysis and represented as a dropout at enrollment).

As illustrated in Figure 1, caregiver enrollment was a two-phase process. In the first phase, eligible caregivers received Kids SIPsmartER recruitment handouts, letters from the principals, and phone calls requesting their participation. Interested caregivers provided written or verbal consent. This was followed by completion of a baseline written or electronic survey for which caregivers received a $10 gift card incentive.

In the second phase, caregivers who consented to the study and completed the baseline survey from the first phase, received an SMS Baseline Assessment. This assessment collected the frequency in which they and their child consumed SSB. Multiple strategies were used to encourage completion of this assessment. Non-responders were given two opportunities to complete baseline data and enroll in the intervention. In the first opportunity, up to three SMS reminder messages were sent. Caregivers had 48 h to respond to the original and reminder messages. For caregivers who had a working phone number but did not complete the SMS Baseline Assessment, a research coordinator called to facilitate the assessment. Non-responders who were not reached by the research coordinator or who were identified as having phone issues were mailed a letter encouraging them to call the research team to complete the SMS Baseline Assessment or provide a new phone number. In the second opportunity, the SMS Baseline Assessment was resent to non-responders at the same time as enrolled caregivers received Assessment 2. This second attempt utilized two SMS reminder messages and 48 h response durations to encourage completion of enrollment.

Caregivers were not enrolled or were removed from enrollment in the SMS intervention if they: (1) did not complete the SMS Baseline Assessment after two attempts, (2) had unresolved phone issues that interfered with receipt of SMS messages (e.g., disconnected mobile number, land line), or (3) actively requested removal from SMS messages (active dropout).

### 2.3. Intervention

**Figure 2:**
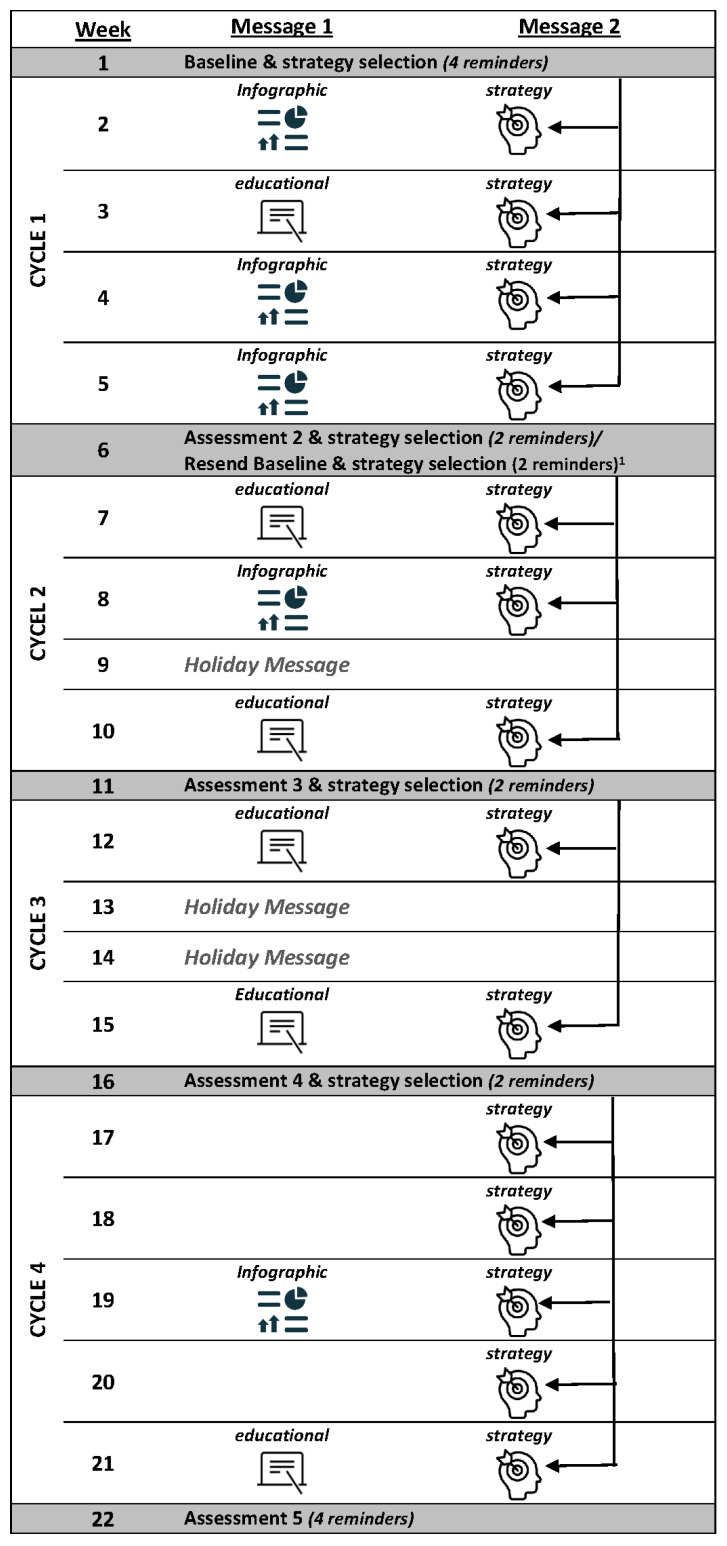
Sample caregiver SMS intervention implementation process. ^1^ Baseline Assessment is resent at the time of Assessment 2 for those who did not respond to the original Baseline Assessment. Intake responses for these caregivers serve for both Baseline and Assessment 2.

As part of the caregiver intervention, consented and surveyed participants were oriented to Kids SIPsmartER through receipt of a newsletter that contained information on SSBs, consumption health risks and recommendations, nutrition labels, and reduction strategies. The SMS intervention that followed consisted of 12 SSB educational messages, 14 strategy messages to overcome SSB reduction barriers, and a series of five assessment messages to measure changes in caregiver and child SSB consumption (see Figure 2). Assessment messages use a one-item question adapted from the Beverage Intake Questionnaire (BEVQ-15) [31] to assess frequency of SSB intake. Caregivers reported SSB intake in Baseline through Assessment 4. Those who consumed SSB or had a consuming child, were given the opportunity to select a strategy message that corresponded to a personal SSB reduction barrier (e.g., parenting tips, tasty alternatives, breaking habits, home and shopping tips, and dealing with friends and family). Tailored feedback on SSB intake changes for caregivers and children were provided for Assessments 2 through 5. In between assessment messages, caregivers received weekly educational messages that summarized the information taught in the classroom component of Kids SIPsmartER. Some of the educational messages were accompanied by an external link provided at the end of a text message. The link redirected the caregiver to an infographic that opened in their mobile phone’s browser. Along with the weekly educational message, caregivers also received a weekly strategy message under the topic they chose during the preceding assessment. All assessment messages and many of the educational and strategy messages were personalized with the names of either or both the caregiver and their child (see Supplementary Table S1 for sample messages).

### 2.4. Implementation Processes

The Qualtrics Research Suite was used to deliver all caregiver SMS messages. As informed through pilot testing [32], SMS messages were delivered over four cycles that were 4 to 5 weeks long. Each cycle began with a two-way assessment message to determine caregiver and child SSB consumption frequency for the prior week and to establish which personalized SSB strategy topic caregivers wanted to receive for the subsequent cycle (Figure 2). When both caregiver and child were identified as non-SSB consumers (consumed one or fewer SSBs in a week), the caregiver received positive reinforcement messages instead of the strategy messages. When caregivers did not respond to the assessment or to the strategy selection item, they received a randomly selected strategy. Reminders to encourage assessment completion were sent at 48 h intervals to caregivers who had not responded. For Assessments 2–4, up to two SMS reminders were sent. For Assessment 5, this increased to three SMS reminders and one phone call. Once assessments were completed, cycle messages began. As illustrated in Figure 2, caregivers received combinations of one-way educational messages, infographics, or personalized strategies/positive reinforcement messages to their mobile phones for a maximum of two instances per week. Strategy topics chosen at each assessment determined the strategy messages for all four to five weeks in the cycle.

### 2.5. Data Analysis

SPSS Version 28.0 was used to conduct all data analyses. Frequencies and descriptive statistics (i.e., means and standard deviations) were used to quantify caregiver enrollment, retention, and engagement with SMS messages. As described above, full enrollment in the SMS intervention was defined as completing consent, the Baseline Survey, and the SMS Baseline Assessment. Individual school enrollment rates were determined by dividing caregiver enrollment by total potential caregivers within the given school year. Retention was defined as the proportion of fully enrolled caregivers who completed Assessment 5.

Engagement with SMS messages was measured as completion of assessments, strategy message selection, and viewing educational infographic messages (collecting open and read rates for education and strategy messages was not possible). Assessment completion was examined for the proportion of enrolled caregivers who reported SSB intake for themselves and their child. These data were further analyzed for proportional increases by each reminder sent. Strategy selection was examined for the proportion of enrolled caregivers who completed the selection process and for the choices they made. Finally, engagement with the infographic messages was calculated as the proportion of enrolled caregivers who clicked on the provided URL to open each message.

To examine pre–post changes in SSB consumption for caregivers and children, weekly SSB frequency reports for Baseline and Assessment 5 were divided by seven to create a continuous daily intake rate. To account for the nesting feature of the data (i.e., individuals were clustered within cohorts), statistical tests of SSB changes between assessments (both for the total sample and for the sub-sample of SSB consumers with intakes of two or more sugary drinks per week) were conducted using a mixed effect modeling framework. The resulting Wald test accounts for cohort-level cluster robust inference. Effect sizes (i.e., Cohens *d*) were reported to determine the strength of associations. 

## 3. Results

### 3.1. Caregiver Enrollment, Demographics, and Retention

As illustrated in Figure 1, of the 1873 invited caregivers, 602 (32%) consented to the study and completed a Baseline Survey (enrollment, Phase 1). Subsequently, 542 completed Phase 2 of the enrollment process by responding to the SMS Baseline Assessment, for a final enrollment rate of 29%. Of the 60 caregivers who did not complete Phase 2 of the enrollment, most were passive dropouts (*n* = 37, 62%). Others had phone issues (*n* = 16, 27%) or were active dropouts (*n* = 7, 12%). SMS reminder messages and research coordinator calls during the opportunity to complete the SMS Baseline Assessment increased caregiver enrollment by 40%. The second opportunity to complete the SMS Baseline Assessment added 3% of caregivers to the study (see Figure 1). Additional enrollment rates by schools across the four years of the study are provided in Supplementary Figure S1. As seen from totals at each year, there was a drop in enrollment rates between the first two years and the last two years (2018–2019 = 36%, 2019–2020 = 36% versus 2020–2021 = 23%, 2021–2022 = 22%). Additionally, overall comparisons of enrollment rates for schools in their second year of participation in the intervention were slightly higher (32%) than in the first year of participation (27%).

The majority of the 542 enrolled caregivers were female (91%), non-Hispanic White (98%), married (70%), and between the ages of 32 and 48 (82%, mean = 41). Most had at least some college education (70%). With regards to annual household income, 19% had less than USD 25,000, 19% had between USD 25,000 and 50,000, and 62% had above USD 50,000. While most caregivers were employed full-time (55%) or part-time (11%), 6% reported being unemployed and 31% reported being out of the workforce. Additionally, less than one half of caregivers reported being enrolled in government assistant programs such as the Supplemental Nutrition Assistance Program (21%) or Medicaid/State Children’s Health Insurance Program (43%).

As seen in Figure 1, the overall retention rate was 84% (i.e., 454 of the 542 enrolled caregivers who completed Assessment 5). Most caregivers (56%) responded to the original SMS Assessment 5 message. SMS reminders and coordinator calls increased retention by 28% (first reminder = 12%; second reminder = 6%, third reminder = 6%; coordinator call = 4%). Retention rates by schools across the four years of the study are reported in Supplementary Figure S2. Total retention rates across the four years fluctuated very little (between 84% and 88%); however, more caregivers were retained in the intervention through Assessment 5 if they enrolled in the school’s first year of participation in the intervention (87%) as compared to the second year (80%).

### 3.2. Enrolled Caregiver Engagement with SMS Intervention Messages

#### 3.2.1. SMS Intervention Assessment Completion: Assessments 2 through 5

**Figure 3:**
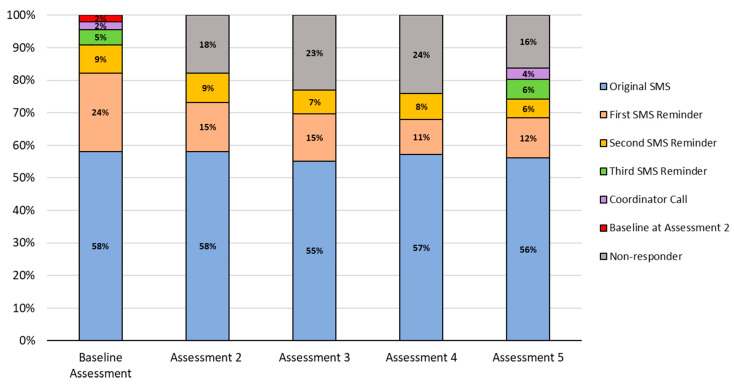
Enrolled caregiver (*n* = 542) response rates by reminders ^1^ for SMS Baseline ^2^ through Assessment 5. ^1^ All assessments included two SMS reminder messages; a third SMS reminder, followed by coordinator call was protocol only for Baseline and Assessment 5. ^2^ For the 14 (3%) caregivers that completed Baseline Assessment at the second attempt (during Assessment 2), their response rates are included in the Assessment 2 data (*n* = 10 responded to the original SMS and *n* = 2 responded to the second SMS reminder).

As seen in Figure 3 completion of Assessments 2–5 was high, ranging from 76% to 84%. Of those enrolled, 63% completed all four of the assessments, while 14% completed three, 7% completed two, 10% completed one, and 6% completed zero of the four assessments. Overall, SMS reminders and coordinator calls to caregivers to complete assessments helped to improve response rates by 19–40%. Most of this improvement was from the first two SMS reminders (range of increase 18–33%). As described earlier, reminders at Baseline and Assessment 5 improved enrollment and retention by 41% and 28%, respectively.

Averaged across all reminders and at each assessment point, most enrolled caregivers (97%) who responded via a replied SMS, did so within the first 24 h (Baseline = 504/529 (95%), Assessment 2 = 426/445 (96%), Assessment 3 = 411/417 (99%), Assessment 4 = 399/409 (98%), Assessment 5 = 417/435 (96%)).

#### 3.2.2. Caregiver Strategy Message Selection: Topics Related to Barriers for Reducing SSB

**Figure 4:**
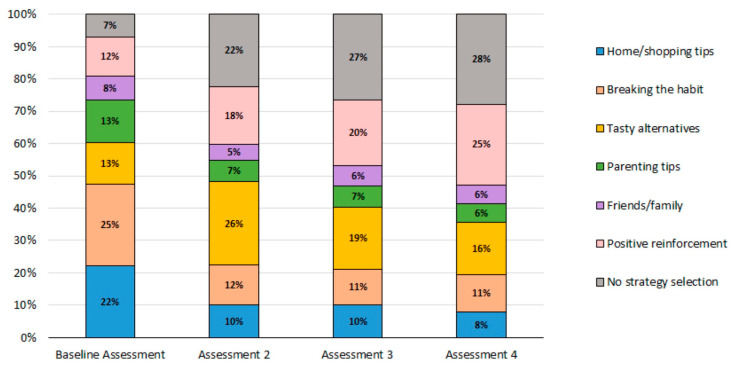
Strategy selections made by enrolled caregivers (*n* = 542) from Baseline ^1^ through Assessment 4 ^2^. ^1^ At the Baseline Assessment, 14 (3%) of the “no strategy selection” caregivers were those who completed the Baseline Assessment at the second attempt (during Assessment 2), and their strategy selections are included in the Assessment 2 data (Home/shopping tips = 3, Breaking the habit = 4, Tasty alternatives = 1, Parenting tips = 2, Friends/family = 1, Positive reinforcement = 2, Partial responder = 1). ^2^ Caregivers do not select a strategy at Assessment 5.

Overall, 57% of caregivers selected a strategy topic for all assessments from Baseline through Assessment 4. The remaining 43% of caregivers selected strategy topics at three (19%), two (11%), one (12%), or none (2%) of the assessments. Figure 4 highlights the selection and shift in barrier topics across the assessments. As shown, the most selected strategies were “tasty alternatives” and “breaking the habit.” Strategies related to “friends and family” were selected the least. Non-consumers did not select strategies and rather received positive reinforcement messages (12–25% across assessments). Lastly, 7–28% of caregivers received coordinator selected strategies as they did not (fully) complete the assessment.

#### 3.2.3. Caregiver Engagement with Educational Infographic Messages

Table 1 shows the five educational infographic messages, accompanying image, and link click rates accumulated across the four years of the study. Overall, the average click rate was around 28%. The link for the Lesson 1 infographic explaining sugary drinks was the most clicked on by enrolled caregivers (37%). Of the 542 enrolled caregivers that were retained, 45% never clicked on any of the infographics, 21% clicked on one, 11% on two, 7% on three, 8% on four, and 9% clicked on all five.

### 3.3. Changes in SSB Intake Frequency

As seen from Table 2, daily SSB frequency significantly (*p* < 0.01) declined between Baseline and Assessment 5 for caregivers (−0.32 (0.03), effect size (ES) = 0.51) and children (−0.26 (0.01), ES = 0.53), while accounting for individuals clustered within cohorts. Similarly, comparisons between a sub-sample of SSB consuming caregivers and children (i.e., consumed two or more SSBs per week at Baseline Assessment) revealed a significant decline in daily SSB frequency. Effect sizes were larger when limited to consumers only (i.e., caregivers ES = 0.65, children ES = 0.67).

## 4. Discussion

Overall, evidence demonstrates the promise of an SMS intervention to engage rural caregivers and to support SSB reduction goals for caregivers and their adolescent child. These findings help fill a gap in the literature addressing enrollment, retention, engagement, and behavioral outcomes among rural caregivers in a school-based SMS SSB reduction intervention [28,29]. Findings may have further implications for school-based SMS interventions targeting other health behaviors.

The high level of caregiver interaction with our SMS assessment messages is encouraging. Overall engagement with these messages ranged from 76% to 84% for reporting SSB intake frequency and from 63% to 92% for selecting a personalized strategy. This high engagement is consistent with other SMS caregiver interventions [19,20] and with reviews that link message tailoring, personalization, and interactivity with higher engagement [12,24,33]. Furthermore, the comparability of these findings with other large studies in urban settings confirms the feasibility of SMS driven interventions within rural regions [33,34].

Additional strategies to bolster engagement with the two-way SMS messages included up to three SMS reminders and one coordinator call. Overall, most caregivers (55% to 58%) completed the assessment on the original text; however, there was a 19% to 40% improvement in completion with the reminders, with 18% to 38% responding within the first two SMS reminders. This finding provides additional evidence for the importance of reminders to engage participants in SMS interventions [25] while suggesting ways to streamline this strategy. For example, the additional coordinator call at Baseline and Assessment 5 yielded relatively few gains in enrollment and retention rates yet was the most costly and burdensome of the strategies. As such, this resource intensive strategy may be unnecessary for future dissemination. This finding may have implications for streamlining other SMS studies.

In contrast to the high levels of engagement with the two-way assessment messages, one-way SMS messages where caregivers were instructed to click on a URL to open an infographic were found to be less engaging. The average views for any one infographic were only 28% and almost one-half (45%) of the caregivers did not open any of the infographics. Similar findings were reported in a study of breastfeeding mothers who were enrolled in the Special Supplemental Nutrition Program for Women, Infants, and Children (WIC). Between 20% to 24% of these women opened their received multimedia messages [35]. This low pattern of engagement may suggest participants did not need visual infographics to support the educational message or it could be the result of using a URL link within an SMS message (e.g., mechanics of clicking the link, lack of trust in clicking an embedded link). For instance, another study that encouraged positive parent–child engagement around literacy and language development reported that most parents either never saw or opened the sent infographic links [36]. Collectively, these findings suggest the need for qualitative studies to determine the acceptability and preferences of different SMS message types as well as process evaluations to examine technologies to bypass the need for embedded links and identify strategies to encourage content engagement.

When examining behavioral impacts, it is important to compare the estimated effect sizes of our SMS intervention to other SSB behavioral and SMS-based interventions. In a recent meta-analysis of randomized controlled trials (RCTs) and interventions to reduce SSB, the effect sizes for SSB reductions were only 0.07 (*p* = 0.16) in the twelve reviewed adult studies and 0.05 (*p* = 0.04) in the five reviewed adolescent studies [28]. Two other seminal meta-analyses on SMS interventions across a wide range of behaviors have found an aggregated effect sizes of 0.39 (*p* < 0.001) among 19 RCTs [12] and 0.24 (*p* < 0.001) among 35 studies [37] of pre–post design (with or without a control group). Our SMS-generated process data presented in this paper is limited by lack of a control condition and hence, effect size comparisons should be cautiously interpreted. Nonetheless, the statistically significant and medium effect sizes among all consumers (i.e., caregivers = 0.51, children = 0.53) and increased effect size among the sub-sample of SSB consumers (caregiver = 0.65, children = 0.67) highlights the promise of our SMS intervention as a primary prevention strategy to target SSB behaviors. When outcome data are available from our larger Kids SIPsmartER cluster RCT [30], more direct effect size comparisons to other RCTs will be important.

In addition to comparison to meta-analyses, two other known studies have applied an SMS intervention strategy to target SSB reduction. A four-week pilot intervention of newsletters and text messages found significant pre–post improvement in the SSB consumption patterns among both parents and children [27]. Similarly, a pilot RCT found significant child SSB improvements among parents randomized to receive an SSB intervention that included text messages [34]. Our behavioral outcome findings are largely congruent with these two studies, while also filling gaps in the literature on school-based SSB interventions targeting caregivers of adolescents [38].

It is important to compare our 27% caregiver enrollment and 84% retention rates with other published findings. Unfortunately, literature gaps on middle school-based caregiver focused SMS interventions limits our ability to directly compare to other similar studies [26]. However, a review of process evaluations from 26 school-based health promotion interventions using more traditional face-to-face methods promoted engagement rates ranging from 33% to 50% [39]. In another study targeting 23 low-income urban elementary schools in a parent SMS program to improve child nutrition, the enrollment rate across four school years ranged between 25% and 35% [40]. These comparisons provide some initial evidence that our SMS caregiver intervention has similar enrollment rates to other face-to-face and SMS school-based interventions. This finding is important because parent participation in school-based health promotion programs has been consistently identified as the least successful and most challenging element, especially in low socio-economic areas [39]. In addition, caregiver participation presumably becomes increasingly challenging as students transition from elementary school into middle and high school. Attempts to ameliorate parent burden for engaging in school-based programming is challenging as it may result in reductions in intervention intensity that can reduce or eliminate behavioral impacts [39]. In contrast, our use of SMS technology to deliver the Kids SIPsmartER caregiver intervention may have struck a balance between caregiver burden and intervention intensity. The high retention rate (84%) for caregivers in our intervention also suggests that messaging remained relevant to the caregivers, and they did not experience study fatigue that may arise from more intense programming.

Due to the timing of our trial, it is important to recognize the potential impacts of COVID-19 on caregiver participation. While adaptations to the technology-based SMS intervention were not needed, schools had to adjust how they distributed and collected enrollment materials to reflect school mitigation plans. Caregiver enrollment dropped for the 2020–2021 and 2021–2022 school years. Notably, the fall enrollment period of both these years was impacted by COVID-19, including widespread school closures that fluctuated week to week. Distribution of study recruitment materials and return of consents were increasingly difficult during these two years. Reviews suggest a decline in overall clinical trial participation and an increase in attrition during COVID-19 [41]. Studies also indicate that parents of school-aged children, particularly from lower income households, were overwhelmed by the sudden shift to remote learning [42,43,44]. Caregivers, inundated with new and increased responsibilities, might have been less willing to join an optional school-based program or to commit to changing health behaviors. Our experience with the Kids SIPsmartER caregiver intervention reflects this literature on enrollment; yet retention rates were relatively consistent before and during COVID-19, suggesting that enrolled caregivers were motivated to participate.

Several limitations of this study should be noted. First, representativeness and generalizability should be considered when interpreting the results. Compared to national demographics, the central regions of Appalachia are largely White, non-Hispanic (90% compared to 60% nationally), with lower educational attainment (28% with at least an associate degree compared to 43% nationally), higher unemployment rate (5.1% compared to 4.4% nationally), and higher poverty rates (19% compared to 13% nationally) [45]. We recognize that the demographic composition of the 71% of caregivers who did not opt-in to the SMS component of Kid’s SIPsmartER is unknown. While demographic data from enrolled participants demonstrate some congruency with regional characteristics, without additional reach data from unenrolled participants, we are unable to fully determine the representativeness of our sample. Furthermore, our study region’s unique demographic characteristics may restrict generalizability of the findings to other populations. Second, since our one-way SMS messages did not require a reply or click on a link, we were unable to capture engagement data from the strategy and non-infographic educational messages. Third, behavioral data rely on caregiver self-reporting, which may be subject to bias. Finally, the lack of a control group somewhat limits comparison with other SSB reduction interventions. In addition, the SMS intervention was intended as a strategy to engage caregivers in the school-based Kids SIPsmartER program. It was outside of the study’s scope to implement and compare an SMS only arm. Therefore, it is not possible to determine the impact of the SMS intervention alone. These limitations should also be interpreted with the study strengths, including a theory-guided SMS intervention with content and structure developed from end users’ input [32], a robust process evaluation of a caregiver focused and school-based SMS intervention, and focus on a rural, medically underserved and lower socio-economic status region with known SSB-related disparities.

Mobile health pilot studies in under resourced communities have shown promising feasibility [13], including the current study, which demonstrated success around engagement strategies and behavioral impacts in a researcher led trial. Future dissemination and implementation studies are critical to understanding how an SMS intervention would perform if scaled up across more schools and more diverse caregivers. Future studies should also explore and refine successful engagement strategies, such as targeting and tailoring SMS messages [12,46]. There is also evidence that using demographic data to target participants at “higher risk” of disengaging can improve intervention efficacy [12]. SMS educational messages could be further tailored to caregivers’ unique SSB situations through systematic evaluation of each assessment to determine if caregiver–child dyads have decreased, increased, or not changed their behavior. This behavioral data could be used to guide promising digital intervention methods, such as just-in-time adaptive intervention or micro-randomization approaches [47]. In addition to expanding engagement strategies for the Kids SIPsmartER caregiver intervention, additional research is necessary to examine how levels of caregiver engagement with the intervention impacted the behavioral outcome. Finally, in alignment with the surge in smartphone ownership among adolescents [48] and a recent meta-analysis of SMS interventions with school aged children [26], future studies should explore the feasibility and efficacy of adding an SMS component for children to enhance the school-based curriculum of the Kids SIPsmartER program.

## 5. Conclusions

Overall, our SMS intervention revealed a relatively high enrollment rate and a retention rate similar to other school-based caregiver interventions. We also found high levels of engagement with SSB behavioral monitoring features and selection of tailored strategy messages. Significant behavioral improvements in self-reported SSB intake for both caregivers and their children were also found, and with greater effect sizes within a subsample of SSB consumers. Equally as important, our study highlights implementation and engagement procedures for researchers and interventionists who may want to replicate an SMS intervention in other regions and across other behavioral contexts. In addition to adding to the nutrition and school-based health promotion literature, our study provides insight into how rural caregivers interact with various SMS messages and demonstrates the practicality of using SMS-based approaches in rural settings.

## Figures and Tables

**Table 1:** Infographic images and enrolled caregiver (*n* = 542) total link click rates.

Infographic	Click Rate	Text Message	Accompanying Image
Lesson 1: Traffic Light	201 (37%)	KidsSIPsmartER: Sugary drinks include: soda/pop, sweet tea, sports drinks, juices, and energy drinks. Think about which ones you drink. Click this secure link to see more! [Image Link]	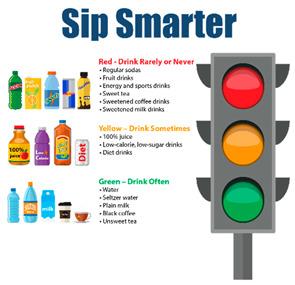
Lesson 3: Health Risks	143 (26%)	KidsSIPsmartER: Trying to be healthy? Drinking fewer sugary drinks can prevent tooth decay, weight gain, and heart problems. Click this secure link to see all the ways sugary drinks can harm your health. [Image Link]	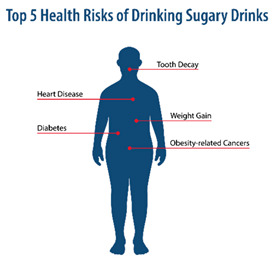
Lesson 4: Nutrition Facts Label	128 (24%)	KidsSIPsmartER: The nutrition label helps you find grams of sugar. It tells the truth, unlike logos & pics on the front of the bottle. Click this secure link to see a quick guide to help you practice with your family. [Image Link]	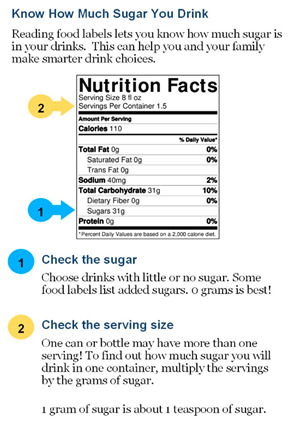
Lesson 6: This or That	153 (28%)	KidsSIPsmartER: Don’t drink your calories! Use your calories to eat more fruits and veggies. Click this secure link to see what you could replace with a sugary drink. [Image Link]	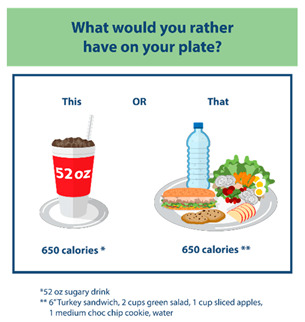
Lesson 11: Saving Money	125 (23%)	KidsSIPsmartER: The cost of sugary drinks really adds up! Spending $1 a day on a pop can add up to $365 over a year. Click this secure link to see how it piles up! [Image Link]	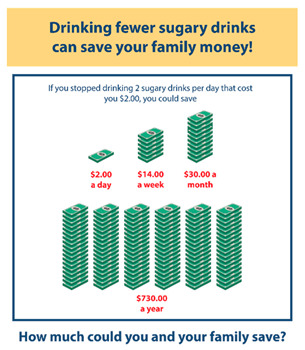

Note: Caregivers who enrolled at the second attempt (during Assessment 2) received Cycle 1 SMS messages.

**Table 2:** Caregiver self-report of personal and child’s sugar-sweetened beverage (SSB) changes from Baseline to Assessment 5.

	*n*	Baseline Daily SSB Frequency ^1^ Mean (SD)	Assessment 5 Daily SSB Frequency Mean (SD)	Mean Change in Daily SSB Frequency (SE)	Effect Size	Wald Test Statistics
All caregivers and children						
Caregiver intake (times/day)	454	0.64 (0.71)	0.33 (0.51)	−0.32 (0.03)	d = 0.51	12.21 *
Child intake (times/day)	454	0.54 (0.50)	0.28 (0.34)	−0.26 (0.02)	d = 0.53	10.79 *
Sub-sample of SSB consumers at Baseline ^2^						
Caregiver intake (times/day)	325	0.87 (0.73)	0.42 (0.57)	−0.45 (0.04)	d = 0.65	14.86 *
Child intake (times/day)	344	0.69 (0.48)	0.34 (0.36)	−0.35 (0.03)	d = 0.67	12.27 *

Note: * indicates statistical significance at *p* < 0.001. ^1^ SMS Baseline Assessment includes the SSB intake responses for the 14 (3%) caregivers that completed Baseline Assessment at the second attempt (during Assessment 2). ^2^ Caregiver and child SSB consumers were defined as those who consumed two or more SSBs per week at SMS Baseline Assessment.

## Data Availability

Study is currently ongoing. Questions about access to data supporting these results may be directed to the corresponding author, Jamie Zoellner.

## Supplementary Materials

The following supporting information can be downloaded at: https://www.mdpi.com/article/10.3390/nu15081957/s1, Table S1: Sample SMS messages; Figure S1: Caregiver SMS enrollment rates by school, year, and year of participation in the study (*n* = 542); Figure S2: Retention rates by school, year, and year of participation in the study (*n* = 542).

## References

[1] Rosinger A., Herrick K.A., Gahche J.J., Park S. (2017). Sugar-Sweetened Beverage Consumption among US Youth, 2011–2014. NCHS Data Brief.

[2] Lane H., Porter K.J., Hecht E., Harris P., Kraak V., Zoellner J. (2018). Kids SIPsmartER: A feasibility study to reduce sugar-sweetened beverage consumption among middle school youth in Central Appalachia. Am. J. Health Promot..

[3] Zoellner J., Estabrooks P.A., Davy B.M., Chen Y.-C.-Y., You W. (2012). Exploring the theory of planned behavior to explain sugar-sweetened beverage consumption. J. Nutr. Educ. Behav..

[4] Malik V.S., Pan A., Willett W.C., Hu F.B. (2013). Sugar-sweetened beverages and weight gain in children and adults: A systematic review and meta-analysis. Am. J. Clin. Nutr..

[5] Imamura F., O’Connor L., Ye Z., Mursu J., Hayashino Y., Bhupathiraju S.N., Forouhi N.G. (2015). Consumption of sugar sweetened beverages, artificially sweetened beverages, and fruit juice and incidence of type 2 diabetes: Systematic review, meta-analysis, and estimation of population attributable fraction. Br. Med. J..

[6] Cheungpasitporn W., Thongprayoon C., Edmonds P.J., Srivali N., Ungprasert P., Kittanamongkolchai W., Erickson S.B. (2015). Sugar and artificially sweetened soda consumption linked to hypertension: A systematic review and meta-analysis. Clin. Exp. Hypertens..

[7] Bernabé E., Vehkalahti M.M., Sheiham A., Aromaa A., Suominen A.L. (2014). Sugar-sweetened beverages and dental caries in adults: A 4-year prospective study. J. Dent..

[8] Yuhas M., Porter K.J., Hedrick V., Zoellner J.M. (2020). Using a socioecological approach to identify factors associated with adolescent sugar-sweetened beverage intake. J. Acad. Nutr. Diet..

[9] Brown R., Ogden J. (2004). Children’s eating attitudes and behaviour: A study of the modelling and control theories of parental influence. Health Educ. Res..

[10] Watts A.W., Miller J., Larson N.I., Eisenberg M.E., Story M.T., Neumark-Sztainer D. (2018). Multicontextual correlates of adolescent sugar-sweetened beverage intake. Eat. Behav..

[11] Thomas T.L., DiClemente R., Snell S. (2014). Overcoming the triad of rural health disparities: How local culture, lack of economic opportunity, and geographic location instigate health disparities. Health Educ. J..

[12] Head K.J., Noar S.M., Iannarino N.T., Harrington N.G. (2013). Efficacy of text messaging-based interventions for health promotion: A meta-analysis. Soc. Sci. Med..

[13] Anderson-Lewis C., Darville G., Mercado R.E., Howell S., Di Maggio S. (2018). mHealth technology use and implications in historically underserved and minority populations in the United States: Systematic literature review. JMIR mHealth uHealth.

[14] Brewer L.C., Fortuna K.L., Jones C., Walker R., Hayes S.N., Patten C.A., Cooper L.A. (2020). Back to the future: Achieving health equity through health informatics and digital health. JMIR mHealth uHealth.

[15] Militello L.K., Kelly S.A., Melnyk B.M. (2012). Systematic review of text-messaging interventions to promote healthy behaviors in pediatric and adolescent populations: Implications for clinical practice and research. Worldviews Evid. Based Nurs..

[16] Militello L., Melnyk B.M., Hekler E.B., Small L., Jacobson D., Shepherd M., Chung C.-F., Evans W., Fanning J. (2016). Automated behavioral text messaging and face-to-face intervention for parents of overweight or obese preschool children: Results from a pilot study. JMIR mHealth uHealth.

[17] Chu J.T.W., Wadham A., Jiang Y., Whittaker R., Stasiak K., Shepherd M., Bullen C. (2019). Effect of MyTeen SMS-based mobile intervention for parents of adolescents: A randomized clinical trial. JAMA Netw..

[18] Callender C., Thompson D. (2018). Family TXT: Feasibility and acceptability of a mHealth obesity prevention program for parents of pre-adolescent African American girls. Children.

[19] Brown B., Harris K., Dybdal L., Malich J., Bodnar B., Hall E. (2019). Feasibility of text messaging to promote child health in a rural community on an American Indian reservation. Health Educ. J..

[20] Aldoory L., Yaros R.A., Prado A.A., Roberts E., Briones R.L. (2016). Piloting Health text messages for rural low-income mothers: Effects of source similarity and simple action steps. Health Promot. Pract..

[21] Hong Y.A., Cho J. (2017). Has the digital health divide widened? Trends of health-related internet use among older adults from 2003 to 2011. J. Gerontol. B Psychol. Sci. Soc. Sci..

[22] Pew Research Center Mobile Fact Sheet. https://www.pewresearch.org/internet/fact-sheet/mobile/.

[23] Pakter A., Chen L.-L. (2013). The daily text: Increasing parental involvement in education with mobile text messaging. J. Educ. Technol. Syst..

[24] Smith D., Duque L., Huffman J.C., Healy B.C., Celano C.M. (2020). Text message interventions for physical activity: A systematic review and meta-analysis. Am. J. Prev. Med..

[25] Naughton F., Riaz M., Sutton S. (2016). Response parameters for SMS text message assessments among pregnant and general smokers participating in SMS cessation trials. Nicotine Tob. Res..

[26] Fedele D.A., Cushing C.C., Fritz A., Amaro C.M., Ortega A. (2017). Mobile health interventions for improving health outcomes in youth: A meta-analysis. JAMA Pediatr..

[27] Zahid A., Reicks M. (2019). A newsletter/text message intervention promoting beverage-related parenting practices: Pilot test results. Health Promot. Pract..

[28] Vargas-Garcia E.J., Evans C.E.L., Prestwich A., Sykes-Muskett B.J., Hooson J., Cade J.E. (2017). Interventions to reduce consumption of sugar-sweetened beverages or increase water intake: Evidence from a systematic review and meta-analysis. Obes. Rev..

[29] Lane H., Porter K., Estabrooks P., Zoellner J. (2016). A systematic review to assess sugar-sweetened beverage interventions for children and adolescents across the socioecological model. J. Acad. Nutr. Diet..

[30] Zoellner J.M., Porter K.J., You W., Chow P.I., Ritterband L.M., Yuhas M., Loyd A., McCormick B.A., Brock D.-J.P. (2019). Kids SIPsmartER, a cluster randomized controlled trial and multi-level intervention to improve sugar-sweetened beverages behaviors among Appalachian middle-school students: Rationale, design & methods. Contemp. Clin. Trials..

[31] Hedrick V.E., Savla J., Comber D.L., Flack K.D., Estabrooks P.A., Nsiah-Kumi P.A., Ortmeier S., Davy B.M. (2012). Development of a brief questionnaire to assess habitual beverage intake (BEVQ-15): Sugar-sweetened beverages and total beverage energy intake. J. Acad. Nutr. Diet..

[32] Yuhas M., Porter K.J., Brock D.-J.P., Loyd A., A McCormick B., Zoellner J.M. (2019). Development and pilot testing of text messages to help reduce sugar-sweetened beverage intake among rural caregivers and adolescents: Mixed methods study. JMIR mHealth uHealth.

[33] Price S., Ferisin S., Sharifi M., Steinberg D., Bennett G., Wolin K.Y., Horan C., Koziol R., Marshall R., Taveras E.M. (2015). Development and implementation of an interactive text messaging campaign to support behavior change in a childhood obesity randomized controlled trial. J. Health Commun..

[34] Nezami B.T., Ward D.S., Lytle L.A., Ennett S.T., Tate D.F. (2018). A mHealth randomized controlled trial to reduce sugar-sweetened beverage intake in preschool-aged children. Pediatr. Obes..

[35] Martinez-Brockman J.L., Harari N., Perez-Escamilla R. (2017). Lactation advice through texting can help (LATCH): An analysis of intensity of engagement via two-way text messaging. FASEB J..

[36] Pila S., Lauricella A.R., Wartella E. (2019). Using short message (SMS) and multimedia messaging (MMS) to encourage positive parent–child engagement around literacy and language development. Mob. Media Commun..

[37] Armanasco A.A., Miller Y.D., Fjeldsoe B.S., Marshall A.L. (2017). Preventive health behavior change text message interventions: A meta-analysis. Am. J. Prev. Med..

[38] Vézina-Im L.-A., Beaulieu D., Bélanger-Gravel A., Boucher D., Sirois C., Dugas M., Provencher V. (2017). Efficacy of school-based interventions aimed at decreasing sugar-sweetened beverage consumption among adolescents: A systematic review. Public Health Nutr..

[39] Langford R., Bonell C., E Jones H., Campbell R. (2015). Obesity prevention and the Health promoting Schools framework: Essential components and barriers to success. Int. J. Behav. Nutr. Phys. Act..

[40] Grutzmacher S.K., Duru E.B., Speirs K.E., Worthington L., Munger A.L., Lachenmayr L.A. (2018). Using text messages to engage low-income parents in school-based nutrition education. J. Hunger. Environ. Nutr..

[41] Asaad M., Habibullah N.K., Butler C.E. (2020). The impact of COVID-19 on clinical trials. Ann. Surg..

[42] Heers M., Lipps O. (2022). Overwhelmed by learning in lockdown: Effects of COVID-19-enforced homeschooling on parents’ wellbeing. Soc. Indic. Res..

[43] Kalil A., Mayer S., Shah R. (2020). Impact of the COVID-19 Crisis on Family Dynamics in Economically Vulnerable Households.

[44] Garbe A., Ogurlu U., Logan N., Cook P. (2020). COVID-19 and remote learning: Experiences of parents with children during the pandemic. Am. J. Qual. Res..

[45] Pollard K., Jacobsen L.A. (2021). The Appalachian Region: A Data Overview from the 2015–2019 American Community Survey.

[46] Hall A.K., Cole-Lewis H., Bernhardt J.M. (2015). Mobile text messaging for health: A systematic review of reviews. Annu. Rev. Public Health.

[47] Qian T., Walton A.E., Collins L.M., Klasnja P., Lanza S.T., Nahum-Shani I., Rabbi M., Russell M.A., Walton M.A., Yoo H. (2022). The microrandomized trial for developing digital interventions: Experimental design and data analysis considerations. Psychol. Methods.

[48] Rideout V., Peebles A., Mann S., Robb M.B. (2021). Common Sense Census: Media Use by Tweens and Teens.

