# Supplementary material for: Outcomes of a Caregiver-Focused Short Message Service (SMS) Intervention to Reduce Intake of Sugar-Sweetened Beverages in Rural Caregivers and Adolescents
Source: Nutrients. 2023 Apr 19;15(8):1957. doi: 10.3390/nu15081957 (PMC10141983; doi:10.3390/nu15081957)
Supplement: Supplementary file 1 [file nutrients-15-01957-s001.zip › nutrients-2316189-supplementary.pdf]

nutrients

MDPI

*Supplementary Materials*

# Enrollment, Retention, Engagement, and Behavioral Outcomes of a Caregiver-Focused Short Message Service (SMS) Intervention to Reduce Intake of Sugar-Sweetened Beverages in Rural Caregivers and Adolescents

**Donna-Jean P Brock [1,*], Maryam Yuhas [2], Kathleen J Porter [1], Phillip Chow [3], Lee Ritterband [3], Deborah Tate [4] and Jamie M Zoellner [1]**

**Table S1.** Sample SMS messages.

| | | | | |
|---|---|---|---|---|
| | | **SAMPLE ASSESSMENT** | | |
| **Caregiver SSB Intake** | **Child SSB Intake** | **Sample SSB Scenarios[1]** | **Sample Feedback** | **Strategy Selections** |
| **Hi it's KidsSIPSmartER and it's time to do a quick check in! Please answer the next 3 questions. How often did YOU, [CAREGIVER NAME], drink sugary drinks last week? Reply with NUMBER.**<br>[1- less than 1 time]<br>[2- 1 time]<br>[3- 2 to 3 times]<br>[4- 4 to 6 times]<br>[5- Everyday]<br>[6- 2 per day]<br>[7- 3 or more per day] | **How often did [CHILD'S NAME] drink sugary drinks last week? Reply with NUMBER.**<br>[1- less than 1 time]<br>[2- 1 time]<br>[3- 2 to 3 times]<br>[4- 4 to 6 times]<br>[5- Everyday]<br>[6- 2 per day]<br>[7- 3 or more per day] | **1. Caregiver and child non-consumers**<br>**2. Caregiver non-consumer and child reduced**<br>**3. Caregiver non-consuming and child maintained**<br>**4. Caregiver non-consumer and child increased**<br>**5. Caregiver reduced child non-consumer**<br>**6. Caregiver and child reduced**<br>**7. Caregiver reduced and child maintained**<br>**8. Caregiver reduced and child increased**<br>**9. Caregiver maintained and child non-consumer**<br>**10. Caregiver maintained and child reduced** | **1. Caregiver increased and child reduced:**<br>[CHILD'S NAME] has decreased his/her intake since last time, but it seems you have increased your intake from last time. Work together as a family to develop a plan, you can always get back on track!<br><br>**2. Caregiver and child non-consumer:**<br>Congrats! Both you and [CHILD'S NAME] drank little to no sugary drinks last week. A healthy family is a happy family, keep up the awesome work! | **1. Family/Friends tips**<br>**2. Breaking your habit**<br>**3. Home/Shopping tips**<br>**4. Parenting around sugary drinks**<br>**5. Finding tasty alternatives** |
| | | **SAMPLE Educational Messages** | | |
| **Lesson 2** | **Lesson 5** | **Lesson 7** | **Lesson 8** | **Lesson 10** |
| Drink less, live more, throw sugar out the door! Limit sugary drinks to 8oz for adults and 0 for kids to improve health for the whole fam. | Sugary drink companies are good at using ads to convince us to buy their stuff. Don't let these companies fool you! | [CHILD'S NAME] knows why and how to drink fewer sugary drinks. Encourage them to become a leader to help others drink less. | [CHILD'S NAME] created a public service announcement about drinking fewer sugary drinks. Ask how s/he likes being a leader for a healthier community. | Drink less, brush more! Any amount of sugary drinks can cause tooth decay but sipping through the day is worse than drinking it all at once. |
| | | **SAMPLE Strategy Messages[2]** | | |
| **Family/Friends tips** | **Breaking your habit** | **Home/Shopping Tips** | **Parenting around sugary drinks** | **Finding tasty alternatives** |
| Going to a party soon? Plan ahead. Bring your own non-sugary drinks, and share them with your friends. | Wondering where to start breaking your habit? Try keeping a log of when and where you drink sugary drinks. | Next grocery shopping trip, buy half the amount of sugary drinks you normally buy. If drinks finish, that's it till the next trip. | Talk to [your child] about reducing sugary drinks and decide on limits together. Make reducing | Try diluting your sugary drink with ice or water. This will help you reduce your sugary drink |

| | | | |
|---|---|---|---|
| | | sugary drinks a competition & have fun with it | intake AND let you get the taste you crave. |

[1] There are up to 16 possible SSB intake scenarios that combine reducing, maintaining, increasing and not consuming SSBs that have specific SMS messages programmed as feedback to caregivers.

[2] Non-consuming caregiver-child dyads received positive reinforcement messages rather than strategy messages (e.g., Way to go! You and your family are on the healthy lifestyle track! Keep going!).

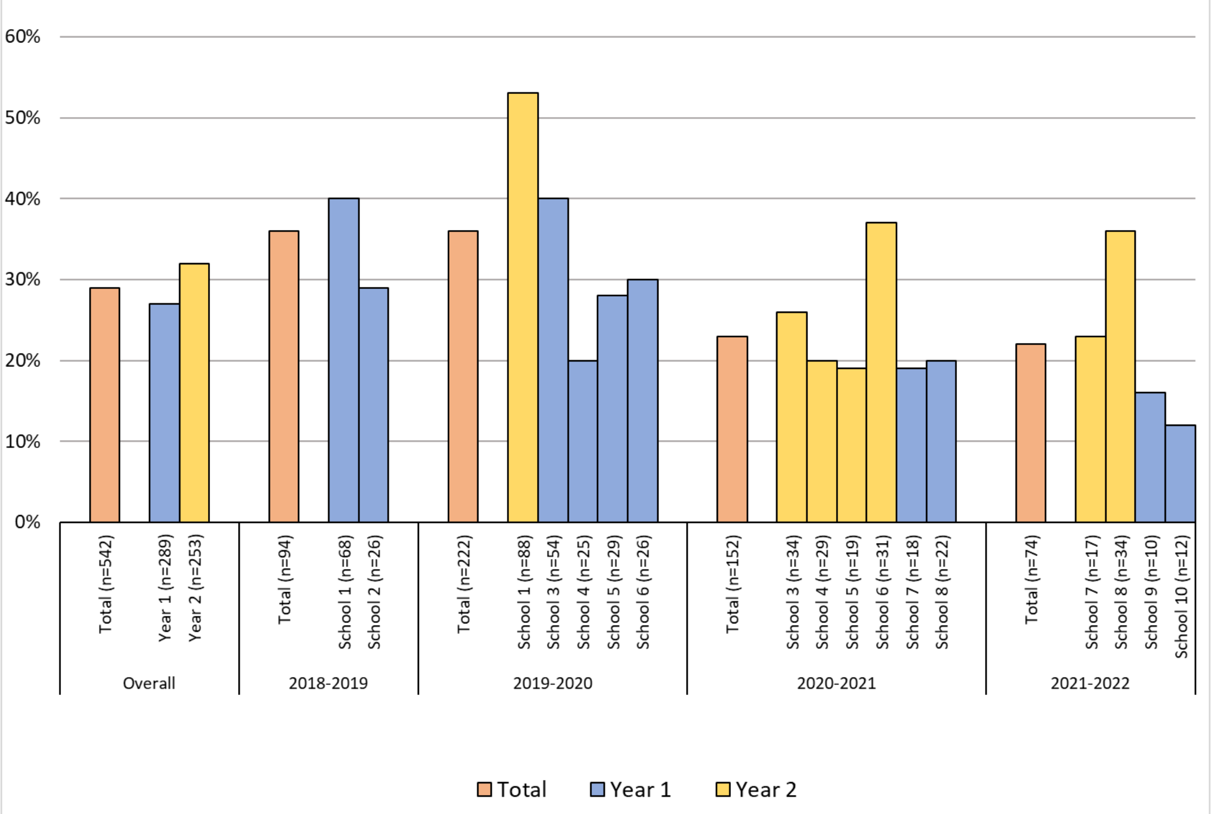


**Figure S1.** Caregiver SMS enrollment rates by school, year, and year of participation in the study (n = 542)[1]. [1]Year of participation in the study refers to year of receipt of the intervention. Some schools were randomized as control schools and received the intervention a year later.

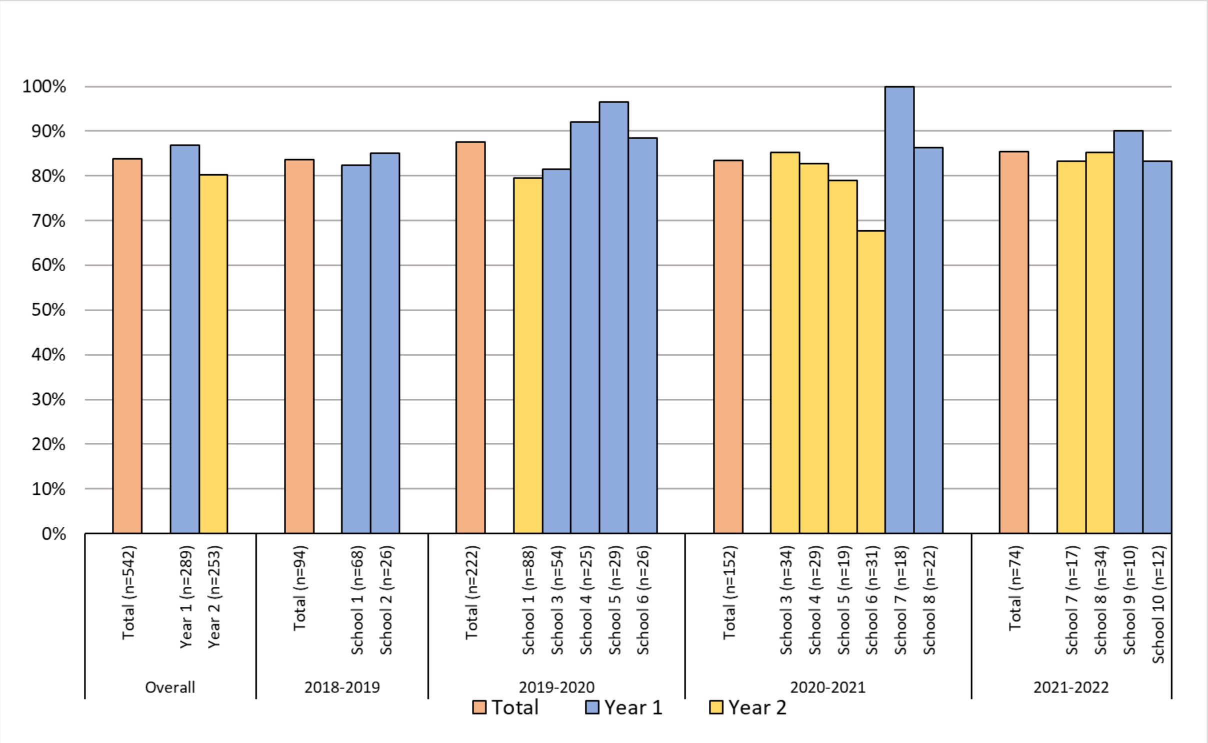


**Figure S2.** Retention rates by school, year, and study phase for enrolled caregivers (n = 542).
